# The Malignant Obstruction Caused by Pancreatic Cancer Within the Uncinate Process in the Third Portion of the Duodenum

**DOI:** 10.7759/cureus.33436

**Published:** 2023-01-06

**Authors:** Teruaki Inoue

**Affiliations:** 1 Department of Internal Medicine, Fujinomiya City General Hospital, Fujinomiya, JPN

**Keywords:** third portion of the duodenum, self-expandable metal stent, uncinate process cancer, duodenal obstruction, pancreatic head cancer

## Abstract

Pancreatic cancer has a poor prognosis, and it often causes duodenal obstruction and obstructive jaundice associated with tumor invasion. Self-expandable metal stent (SEMS) placement is useful for duodenal obstruction. Pancreatic cancer can occur in the uncinate process, which may lead to malignant obstruction in the third portion of the duodenum. However, the upper gastrointestinal endoscope often cannot reach the third portion of the duodenum, and SEMS placement is sometimes difficult. We report a case of successful SEMS placement with a colonoscope for the obstruction of the third portion of the duodenum due to uncinate process cancer.

A 67-year-old Japanese male was referred to our hospital for palliative treatment of unresectable pancreatic cancer. He complained of anorexia and vomiting and was admitted to our hospital. Computed tomography (CT) scans showed the tumor with delayed enhancement in the pancreatic uncinate process. Esophagogastroduodenoscopy (EGD) and gastrografin enema revealed the stenosis caused by tumor invasion in the third portion of the duodenum. The stenosis was thought to cause his symptom. PCFQ260AZ endoscope (Olympus, Tokyo, Japan) was able to reach the stenosis, and a 22 mm × 80 mm uncovered SEMS (Niti-S, Taewoong Medical, Seoul, South Korea) was placed beyond the stenosis. After SEMS placement, his symptoms disappeared.

Uncinate process cancer is located close to the third portion of the duodenum and caused the obstruction there. We should be cautious about this, and a colonoscope is useful for SEMS placement for malignant obstruction in the third portion of the duodenum.

## Introduction

Pancreatic cancer has a poor prognosis and is often diagnosed in an unresectable state. Patients with pancreatic cancer often suffer from duodenal obstruction and obstructive jaundice due to tumor invasion. As for duodenal obstruction, gastrojejunostomy and self-expandable metal stent (SEMS) placement are considered palliative treatments [1]. Pancreatic cancer can arise from the uncinate process, and uncinate process cancer can cause obstruction in the third portion of the duodenum by tumor invasion. However, SEMS placement is sometimes difficult in the third portion of the duodenum. We report a case of successful SEMS placement with a colonoscope for the malignant obstruction caused by uncinate process cancer in the third portion of the duodenum.

## Case presentation

**Figure 1:**
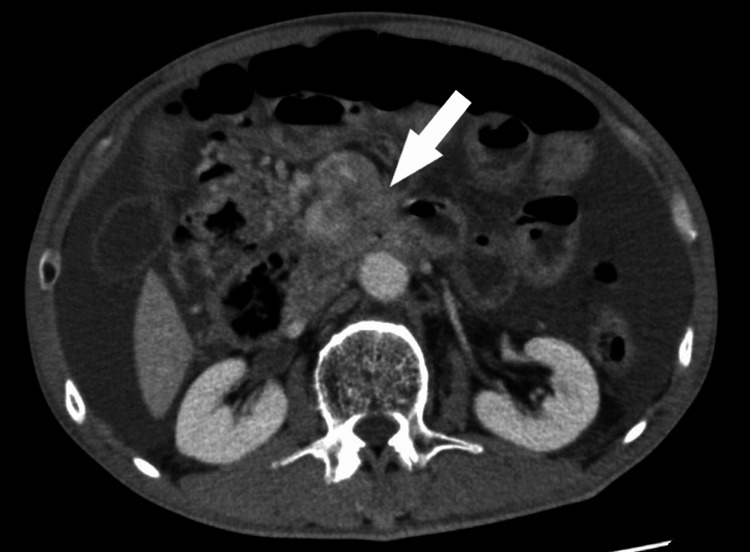
Abdominal contrast-enhanced CT scan (axial view). The tumor with delayed enhancement was confirmed in the pancreatic uncinate process (white arrow). CT: computed tomography

A 67-year-old Japanese male was referred to our hospital for palliative treatment of unresectable pancreatic cancer. He underwent chemotherapy for two years, but the treatment stopped working, and he was put on a course of palliative care. He complained of anorexia and vomiting and was admitted to our hospital. Upon examination, his vital signs were normal. No significant findings were observed in blood tests. Computed tomography (CT) scans showed the tumor with delayed enhancement in the pancreatic uncinate process, which invaded the third portion of the duodenum (Figure 1).

**Figure 2:**
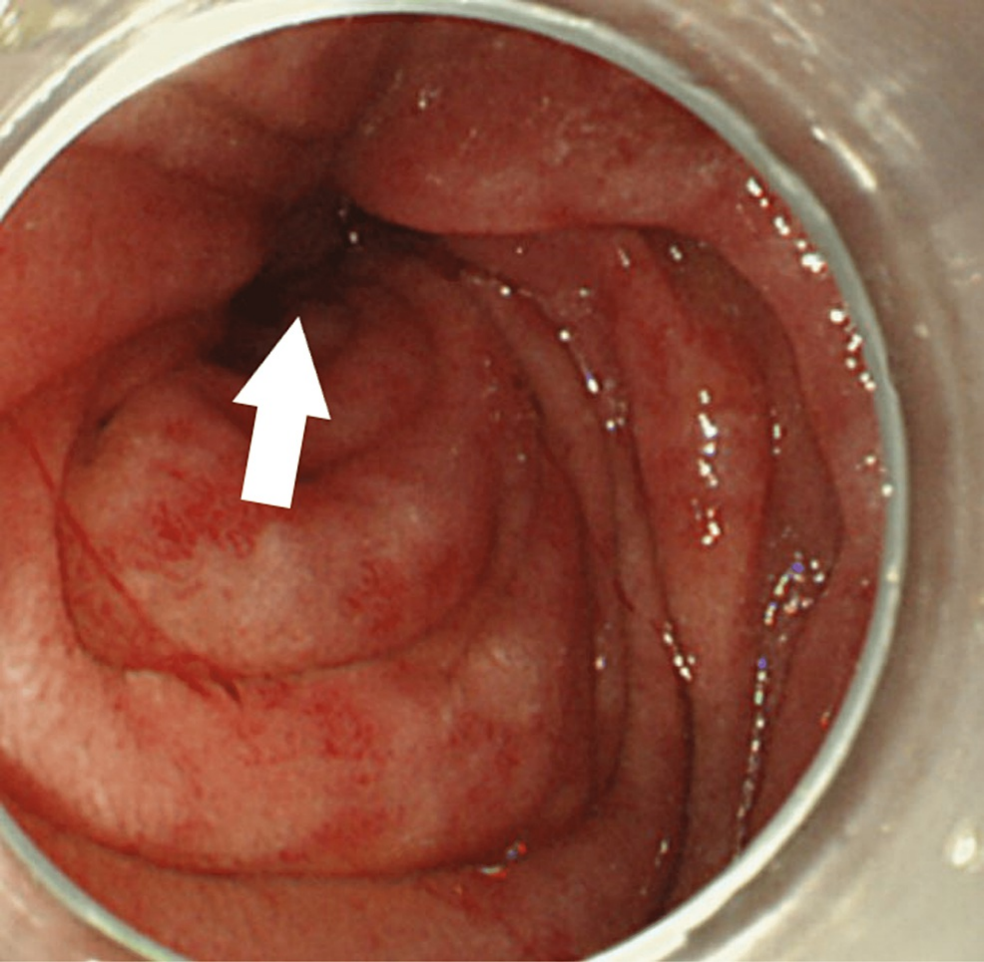
Esophagogastroduodenoscopy finding. Esophagogastroduodenoscopy revealed the mucosal changes, which indicate tumor invasion in the third portion of the duodenum (white arrow).

**Figure 3:**
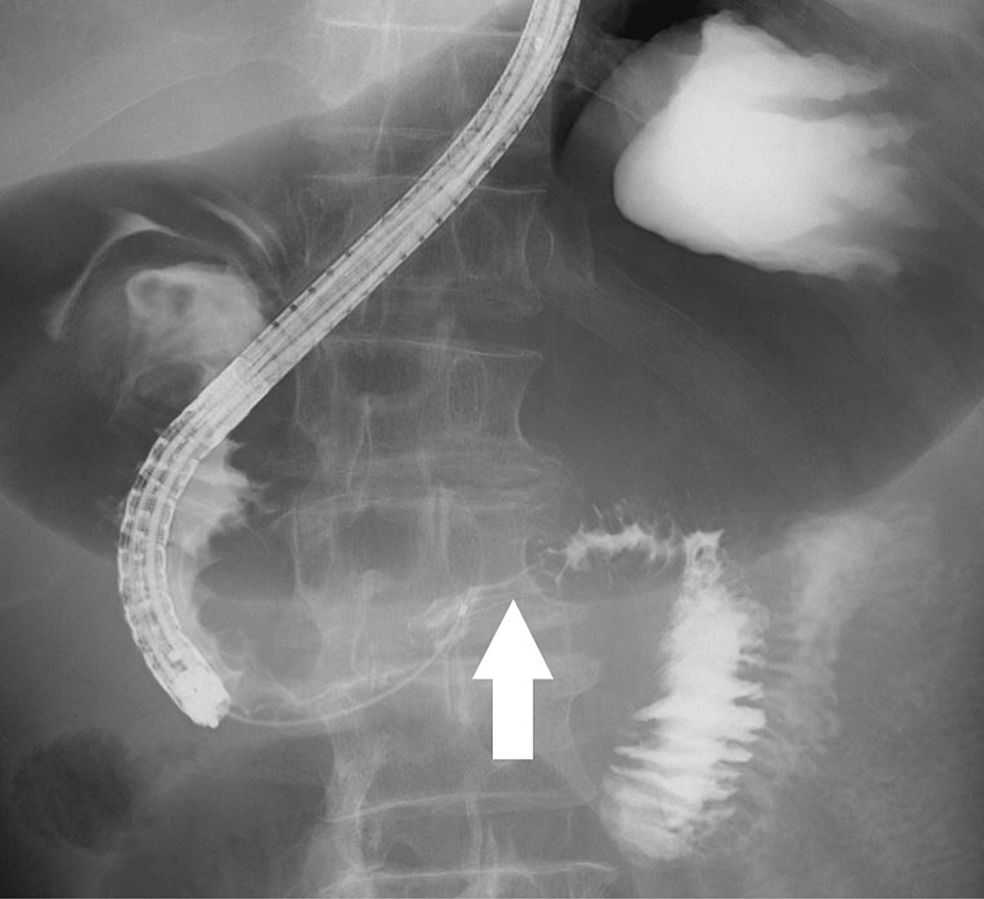
Abdominal X-ray. Gastrografin enema showed the stenosis in the third portion of the duodenum (white arrow).

Esophagogastroduodenoscopy (EGD) and gastrografin enema revealed the stenosis caused by tumor invasion in the third portion of the duodenum (Figure 2 and Figure 3).

**Figure 4:**
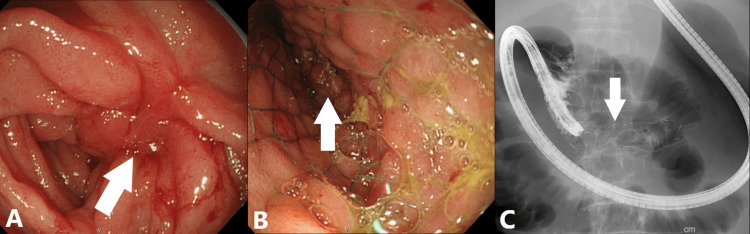
Esophagogastroduodenoscopy finding (A and B) and abdominal X-ray (C). Folds convergence caused by tumor invasion was confirmed in the third portion of the duodenum (A, white arrow). The SEMS was placed beyond the stenosis caused by tumor invasion in the third portion of the duodenum (B and C, white arrow).

Following this, the cause of his symptoms was thought to be duodenal obstruction caused by pancreatic cancer invasion. Although the stenosis was not circumferential, his symptoms were severe, and SEMS placement was considered. However, the GIF-Q260 J endoscope (Olympus, Tokyo, Japan) could not approach the stenosis. Therefore, the PCFQ260AZ endoscope (Olympus, Tokyo, Japan), which is longer than the GIF-Q260 J endoscope, was chosen to place the SEMS. The PCFQ260AZ endoscope was able to reach the stenosis, and a 22 mm × 80 mm uncovered SEMS (Niti-S, Taewoong Medical, Seoul, South Korea) was placed beyond the stenosis (Figure 4A-4C).

After the SEMS placement, his anorexia and vomiting disappeared. He had no recurrence of symptoms after oral ingestion. On the 38th hospital day, he was transferred to another hospital for palliative care.

## Discussion

Pancreatic cancer mainly arises at the head of the pancreas, and its rate is approximately 65% [2]. It often causes several complications such as duodenal obstruction and obstructive jaundice caused by biliary obstruction. Shah et al. have reported that the rate of duodenal obstruction in pancreatic cancer is increasing [3]. Duodenal obstruction can cause vomiting and makes oral intake difficult. Therefore, early intervention is preferred. SEMS placement and gastrojejunostomy are useful in the treatment of duodenal obstruction. SEMS placement is less invasive compared to gastrojejunostomy and is appropriate for patients with poor performance status or short life expectancy. Maire et al. reported that the rate of successful duodenal SEMS placement in unresectable pancreatic cancer was 96%, and the median duration of stent patency was six months [4]. Pancreatic cancer can arise in the region of the uncinate process [5]. The uncinate process is a prolongation of the inferior part in the head of the pancreas, which is a hooklike projection. Uncinate process cancer is rare, and its incidence is 2.5% [6]. Compared to non-uncinate process pancreatic head cancer, uncinate process cancer tends to be less jaundiced [7]. However, uncinate process cancer is located close to the third portion of the duodenum and can cause obstruction there. In our case as well, the patient was not jaundiced but had an obstruction in the third portion of the duodenum.

The upper gastrointestinal endoscope is about 100-110 cm in length, and it often cannot reach the lesion in the third portion of the duodenum. On the other hand, the lower gastrointestinal endoscope is about 130 cm in length, longer than the upper gastrointestinal endoscope. In our case, we were able to reach the lesion and placed the SEMS beyond the stenosis with the lower gastrointestinal endoscope, the PCFQ260AZ endoscope. The lower gastrointestinal endoscope is useful for the lesion in the third portion of the duodenum.

## Conclusions

In summary, we have presented a case of successful SEMS placement with a colonoscope for the malignant obstruction in the third portion of the duodenum caused by uncinate process cancer. Uncinate process cancer often causes malignant obstruction in the third portion of the duodenum. When obstruction occurs, SEMS placement with a colonoscope is useful.
